# Relationship between antibiotic use and short-term risk of mortality in patients with sepsis-associated encephalopathy: a study based on the medical information mart for intensive care database

**DOI:** 10.1186/s12879-025-11139-3

**Published:** 2025-07-01

**Authors:** Shuwen Xiao, Jinqin Li, Qin Zhu, Yan Pu, Junjie Hao, Hanming Gong

**Affiliations:** 1 College of Traditional Chinese Medicine, Yunnan University of Chinese Medicine, 1076 Yuhua Road, Chenggong District, Kunming, Yunnan Province 650500 P.R. China; 2 Department of Pharmacy, Yunnan New Kunhua Hospital, Taian Road, Taiping New District, Anning City, Kunming, Yunnan Province 650301 P.R. China

**Keywords:** Relationship, Antibiotic, Mortality, Sepsis-associated encephalopathy

## Abstract

**Background:**

In critically ill sepsis patients the use of certain antibiotics can be associated with adverse effects. This study investigates the relationship between the use of different classes of antibiotic during the intensive care unit stay and the 30-day mortality risk in patients with sepsis-associated encephalopathy.

**Methods:**

This retrospective observational study collected data from the Medical Information Mart for Intensive Care IV database between 2008 and 2019. The antibiotic classes assessed included cephalosporins, penicillins, carbapenems, quinolones, macrolides, and metronidazole. The Cox proportional hazards model was employed to assess the association between the use of different classes of antibiotic and mortality risk in patients with sepsis-associated encephalopathy.

**Results:**

The 30-day mortality was 16.19% (643 out of 3974 patients). The use of penicillins (hazard ratio: 1.32, 95% confidence interval: 1.11–1.58, *P* = 0.002), macrolides (hazard ratio: 1.50, 95% confidence interval: 1.13–2.00, *P* = 0.005), and metronidazole (hazard ratio: 1.32, 95% confidence interval: 1.11–1.57, *P* = 0.002) were associated with a higher risk of 30-day mortality. The use of one, two, or more than three antibiotic classes were associated with an increased risk of 30-day mortality (all *P* < 0.05). In sepsis-associated encephalopathy patients aged ≥ 65 years, with Sequential Organ Failure Assessment scores ≥ 6, Charlson comorbidity index scores ≥ 2, Glasgow Coma Scale ≥ 8, experiencing acute kidney injury, and receiving opiates or propofol, the number of administered antibiotic classes was significantly associated with increased 30-day mortality risk.

**Conclusion:**

We found an association between penicillins, macrolides, and metronidazole usage and 30-day mortality in sepsis-associated encephalopathy patients that needs future prospective randomized control trials to establish causal relationship.

**Supplementary Information:**

The online version contains supplementary material available at 10.1186/s12879-025-11139-3.

## Background

Sepsis is a life-threatening clinical condition resulting from the host's dysregulated response to infection [1]. Sepsis-associated encephalopathy (SAE) is a serious neurologic syndrome characterized by diffuse brain dysfunction due to sepsis [2, 3]. SAE has a high incidence rate of up to 70% among patients admitted to the intensive care unit (ICU) [4]. SAE is associated with long-term cognitive impairments and focal neurological deficits [5]. Additionally, SAE significantly increases hospital length of stay and short-term mortality risk [6]. Even mild alterations in consciousness can lead to a significant increase in the short-term risk of mortality for patients with sepsis [7]. Identifying factors that influence short-term mortality in patients with SAE is crucial for improving patient outcomes.

Antibiotics are essential in treating sepsis patients, serving as a critical intervention that can improve outcomes including mortality [8, 9]. While antibiotics are indispensable in treating infections, their potential neurotoxic adverse effects may affect outcomes [10, 11]. Previous studies have indicated that the use of certain types of antibiotic may lead to antibiotic-associated encephalopathy, which primarily includes encephalopathy with seizures or myoclonus caused by cephalosporins and penicillins, encephalopathy characterized by psychiatric symptoms induced by quinolones and macrolides, and encephalopathy associated with cerebellar signs and magnetic resonance imaging (MRI) abnormalities caused by metronidazole [12–14]. In critically ill patients, the association between antibiotic use and delirium remains inconsistent. Grahl et al. found that exposure to first- to third-generation cephalosporins was associated with an increased risk of delirium, whereas no significant association was observed with fourth-generation cephalosporins (e.g., cefepime), penicillins, carbapenems, fluoroquinolones, or macrolides [15]. In critically ill sepsis patients, the use of quinolones and carbapenems is a risk factor for the development of SAE [16].

In the context of sepsis management, empirical antibiotic therapy is crucial for early intervention, often involving broad-spectrum antibiotics to cover potential pathogens while awaiting culture results [17, 18]. However, to date, research is lacking regarding the association between different antibiotic classes and the prognosis of critically ill patients who have developed SAE. Given the potential neurotoxicity associated with certain antibiotics, understanding the relationship between antibiotic use and mortality in SAE patients is of significant clinical importance. This study aims to investigate the association between antibiotic use and short-term mortality risk in SAE patients.

## Methods

### Study design and patients

The study was designed as a retrospective observational study with patient enrollment spanning 2008 to 2019. The data source for this study was the Medical Information Mart for Intensive Care (MIMIC)-IV database (https://mimic.mit.edu/docs/iv/). MIMIC-IV is a relational database containing real hospital stays for patients admitted to Beth Israel Deaconess Medical Center (BIDMC), a tertiary academic medical center in Boston, MA, USA. MIMIC-IV contains comprehensive information for each patient while they were in the hospital: laboratory measurements, medications administered, vital signs documented, and so on. The database is intended to support a wide variety of research in healthcare. MIMIC-IV builds upon the success of MIMIC-III and incorporates numerous improvements over MIMIC-III. Patients diagnosed with SAE upon ICU admission in MIMIC-IV were included in this study. The criteria for exclusion in this study were defined as follows: (1) Patients under the age of 18, (2) Patients with an ICU stay shorter than 24 h, (3) Patients who did not receive any antibiotic while in the ICU admission, (4) Patients with a follow-up time of less than one day, (5) Patients with a primary brain injury, including traumatic brain injury, ischemic or hemorrhagic stroke, epilepsy, or intracranial infections, (6) Patients with a history of chronic alcohol or drug abuse, (7) Those with pre-existing liver or kidney conditions that could impair consciousness. As this paper was derived from a publicly available database, no ethical approval was required.

### Data collection

Data collected from the database included the following: (1) baseline characteristics: age (years), gender, race, weight (kg), comorbidity [myocardial infarction, congestive heart failure, chronic pulmonary disease, acute kidney injury (AKI), liver disease, diabetes, and anemia], (2) vital signs: heart rate (bpm), systolic blood pressure (SBP, mmHg), diastolic blood pressure (DBP, mmHg), respiratory rate (bpm), SpO_2_ (%), and temperature (℃), (3) scoring systems:Sequential Organ Failure Assessment (SOFA, score), Glasgow Coma Scale (GCS, score), and Charlson comorbidity index (CCI, score), (4) laboratory parameters: red blood cell distribution width (RDW, %), platelet count (K/uL), white blood cell count (WBC, K/uL), hematocrit (%), blood urea nitrogen (BUN, mg/dL), glucose (mg/dL), bicarbonate (mEq/L), sodium (mEq/L), potassium (mEq/L), chloride (mEq/L), arterial pH, prothrombin time (PT, seconds), partial thromboplastin time (PTT, seconds), and urine output (mL), (5) intervention or drugs: mechanical ventilation, vasopressor, renal replacement therapy (RRT), opiates drug, propofol drug, midazolam drug, and dexmedetomidine drug, (6) antibiotics: cephalosporins, penicillins, carbapenems, quinolones, macrolides, metronidazole, aminoglycosides, polypeptides, tetracyclines, lincosamides, and antifungal agents.

Myocardial infarction was identified by the code under ICD-9 as 410.xx (ranging from 410.0 to 410.9) and under ICD-10 with codes beginning with I21 (from I21.0 to I21.9). Heart failure was collected in ICD-9 with codes from 428.0 to 428.9, and in ICD-10 with codes commencing with I50 (from I50.0 to I50.9). Chronic obstructive pulmonary disease (COPD) was identified in ICD-9 by codes 4660, 490, 4910, 4911, 49120, 49121, 49128, 49129, and in ICD-10 with codes beginning with J44 (from J44.0 to J44.9). Liver disease was identified in ICD-9 by codes 570 to 573, and in ICD-10 by codes from K70 to K77. Diabetes mellitus was identified in ICD-9 with codes starting with 250 (from 250.0 to 250.9), and in ICD-10 with codes beginning with E10, E11, E12, E13, and E14 (from E10.0 to E14.9). Anemia was a hemoglobin level below 12 g/dL for females and below 13 g/dL for males. Laboratory blood parameters were obtained within the first 24 h following ICU admission.

### Definition of SAE

In accordance with the Sepsis 3.0 criteria for diagnosing sepsis, patients were identified as having SAE if they meet either a GCS score of ≤ 14 within the first 24 h of ICU admission, or if their ICD-9 code was 2930, 2931, and their ICD-10 code were F05 [19, 20].

### Assessment of antibiotics use

The antibiotics assessed in this study included cephalosporins, penicillins, carbapenems, quinolones, macrolides, and metronidazole. The number of antibiotic classes referred to as the combination of these six antibiotic classes. For each patient, we identified the specific antibiotic administered. Specifically, a patient would be counted as exposed to penicillins in the penicillins exposure analysis, as exposed to quinolones in the quinolones exposure analysis, and as exposed to two types of antibiotics in the analysis of the number of classes exposure groups. For example, a patient treated with both ceftriaxones and penicillins would be classified as using two antibiotic classes. The maximum number of classes used was six, while the minimum was zero, indicating no antibiotic use. During the study period, BIDMC followed hospital-wide antibiotic stewardship protocols aligned with Infectious Disease Society of America (IDSA) guidelines [21].

### Outcome and follow-up

The outcome of this study was 30-day mortality. The follow-up commencement date was set at the time of the patient's admission to the ICU, and the follow-up conclusion date was defined as 30 days post-ICU admission. The mean duration of follow-up was 26.75 (± 7.95) days.

### Statistical analysis

Measurement data were assessed for normality using skewness and kurtosis methods [22], with absolute values of kurtosis < 10 and skewness < 3 considered indicative of normality. The homogeneity of variances was tested using Levene’s test [23], using a significance level of *P* < 0.05. For measurement data that were normally distributed, the mean and standard deviation [Mean (± SD)] were used for description, and group comparisons were made using the t-test for cases with homogeneous variances, or the t’ test for heterogeneous variances. Non-normal measurement data were described using the median and interquartile range [Median (Q₁, Q₃)], and group comparisons were conducted using the Wilcoxon rank-sum test. Enumeration data were described in terms of the number of cases and the proportion (n (%)), with group comparisons performed using the chi-square test or Fisher’s exact test. If the proportion of missing values for a variable exceeded 20%, the variable was excluded from the dataset. This cutoff is widely used in clinical research to balance data completeness and potential bias. For variables with a missing value proportion below 20%, missing values were imputed using the Random Forest imputation method [24]. To assess the impact of imputation on study outcomes, sensitivity analyses were conducted using both pre- and post-imputation datasets.

Potential covariates associated with 30-day mortality in SAE patients were initially evaluated using univariate Cox proportional hazards models [25]. Variables with *P*-values less than 0.05 were considered significant and included in a subsequent stepwise regression analysis. This process identified the following covariates for inclusion in the multivariable model: age, race, AKI, SOFA score, CCI, weight, heart rate, respiratory rate, SpO₂, RDW, BUN, sodium, chloride, pH, PTT, urine output, ventilation status, vasopressor use, opioid administration, and propofol administration (Supplementary Table 1). Univariate and multivariable Cox proportional hazards model analyses were employed to assess the association between the use of different antibiotic classes during the ICU stay and the short-term mortality risk in patients with SAE. Subgroup analysis was performed by age (< 65, ≥ 65), sex, SOFA (median), CCI (median), AKI, opiates, and propofol. Kaplan–Meier curves were drawn to show the probability of survival of 30 days across different numbers of antibiotic classes. Hazards ratio (HR) and 95% confidence interval (CI) were effect sizes. The confidence level was set at α = 0.05. Data cleaning (including missing value statistics) and missing value imputation were done using Python 3.9.12 (https://docs.python.org/release/3.9.12/whatsnew/changelog.html), and sensitivity analysis, difference comparison, statistical modeling, and plotting were performed by R version 4.3.1 (2023–06–16 ucrt) [26].

## Results

### Patients’ selection and characteristics of included patients

**Fig. 1:**
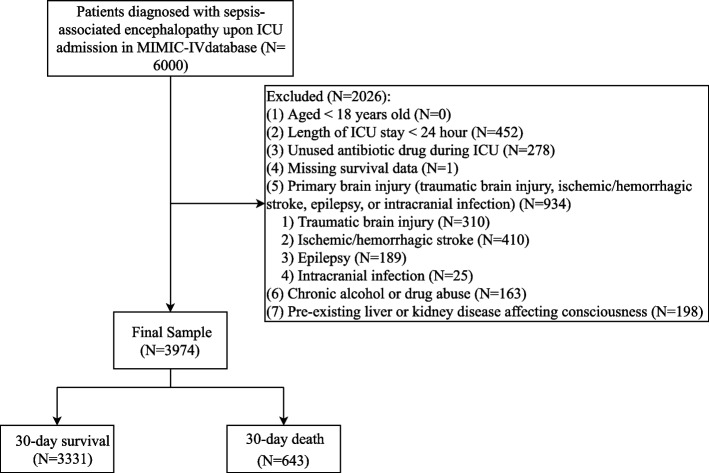
The flow chart of participant selection process

Initially, 6,000 patients diagnosed with SAE upon ICU admission in the MIMIC-IV were identified. Based on the inclusion and exclusion criteria, 3,974 patients were finally included in this study. Of the included patients, 643 (16.19%) patients died within 30 days. Figure 1 depicts the participant selection process. The mean age of the patients was 66.93 years (± 14.86). The gender distribution among the study participants was 2,379 (59.86%) identified as male and 1,595 (40.14%) as female, respectively. Myocardial infarction was reported in 865 (21.77%) of the patients, congestive heart failure was present in 1,312 (33.01%), and AKI was identified in 3,069 (77.23%). Cephalosporins were administered to 2,701 out of 3,974 patients (67.97%). Penicillins was used in 933 out of 3,974 patients (23.48%). Carbapenems were received by 321 out of 3,974 patients (8.08%). Quinolone antibiotics were prescribed to 625 out of 3,974 patients (15.73%). Macrolides were used in 189 out of 3,974 patients (4.76%). Metronidazole was administered to 662 out of 3,974 patients (16.66%). Table 1 summarizes the characteristics of the included patients.

**Table 1:** Characteristics of included patients

Variables	Total (*N* = 3974)	30-day survival (*N* = 3331)	30-day death (*N* = 643)	Statistics	*P*
Length of ICU stay, days, M (Q₁, Q₃)	2.93 (1.62–5.28)	2.66 (1.48–4.98)	4.11 (2.29–7.88)	W = 792,183	< 0.001
Age, years, Mean (± SD)	66.93 (± 14.86)	65.99 (± 14.84)	71.77 (± 14.00)	t = −9.114	< 0.001
Gender, n (%)				χ^2^ = 2.914	0.088
Female	1595 (40.14)	1317 (39.54)	278 (43.23)		
Male	2379 (59.86)	2014 (60.46)	365 (56.77)		
Race, n (%)				χ^2^ = 38.502	< 0.001
Black	272 (6.84)	216 (6.48)	56 (8.71)		
Others	509 (12.81)	433 (13)	76 (11.82)		
Unknown	424 (10.67)	315 (9.46)	109 (16.95)		
White	2769 (69.68)	2367 (71.06)	402 (62.52)		
Myocardial infarct, n (%)				χ^2^ = 10.163	0.001
No	3109 (78.23)	2637 (79.17)	472 (73.41)		
Yes	865 (21.77)	694 (20.83)	171 (26.59)		
Congestive heart failure, n (%)				χ^2^ = 72.902	< 0.001
No	2662 (66.99)	2325 (69.8)	337 (52.41)		
Yes	1312 (33.01)	1006 (30.2)	306 (47.59)		
Chronic pulmonary disease, n (%)				χ^2^ = 26.197	< 0.001
No	2855 (71.84)	2447 (73.46)	408 (63.45)		
Yes	1119 (28.16)	884 (26.54)	235 (36.55)		
AKI, n (%)				χ^2^ = 105.354	< 0.001
No	905 (22.77)	859 (25.79)	46 (7.15)		
Yes	3069 (77.23)	2472 (74.21)	597 (92.85)		
Liver disease, n (%)				χ^2^ = 76.868	< 0.001
No	3589 (90.31)	3069 (92.13)	520 (80.87)		
Yes	385 (9.69)	262 (7.87)	123 (19.13)		
Diabetes, n (%)				χ^2^ = 0.351	0.554
No	2639 (66.41)	2219 (66.62)	420 (65.32)		
Yes	1335 (33.59)	1112 (33.38)	223 (34.68)		
Anemia, n (%)				χ^2^ = 2.397	0.122
No	628 (15.8)	540 (16.21)	88 (13.69)		
Yes	3346 (84.2)	2791 (83.79)	555 (86.31)		
SOFA, score, Mean (± SD)	6.80 (± 3.60)	6.29 (± 3.23)	9.43 (± 4.26)	t'= −17.738	< 0.001
GCS, score, Mean (± SD)	11.23 (± 4.03)	11.36 (± 4.01)	10.55 (± 4.07)	t'= 4.610	< 0.001
CCI, score, Mean (± SD)	2.85 (± 2.34)	2.55 (± 2.19)	4.38 (± 2.51)	t'= −17.188	< 0.001
Weight, kg, Mean (± SD)	82.30 (± 19.49)	83.06 (± 19.50)	78.34 (± 18.97)	t = 5.643	< 0.001
Heart rate, bpm, Mean (± SD)	88.64 (± 18.87)	87.40 (± 18.18)	95.02 (± 20.99)	t'= −8.604	< 0.001
SBP, mmHg, Mean (± SD)	120.21 (± 23.53)	120.88 (± 23.41)	116.73 (± 23.87)	t = 4.110	< 0.001
DBP, mmHg, Mean (± SD)	64.25 (± 15.60)	64.38 (± 15.36)	63.56 (± 16.81)	t'= 1.146	0.252
Respiratory rate, bpm, Mean (± SD)	18.71 (± 6.18)	18.07 (± 5.89)	22.02 (± 6.59)	t'= −14.161	< 0.001
SpO2, %, Mean (± SD)	97.51 (± 3.10)	97.78 (± 2.98)	96.12 (± 3.37)	t'= 11.677	< 0.001
Temperature, ℃, Mean (± SD)	36.57 (± 0.72)	36.56 (± 0.71)	36.60 (± 0.75)	t = −1.204	0.229
RDW, %, Mean (± SD)	15.03 (± 2.28)	14.75 (± 2.06)	16.50 (± 2.74)	t'= −15.467	< 0.001
Platelet, K/uL, Mean (± SD)	204.10 (± 110.81)	202.84 (± 106.43)	210.66 (± 131.12)	t'= −1.425	0.154
WBC, K/uL, M (Q₁, Q₃)	12 (8.8–16.3)	11.9 (8.8–15.9)	12.8 (8.9–18.25)	W = 968,141	< 0.001
Hematocrit, %, Mean (± SD)	31.00 (± 6.52)	30.99 (± 6.48)	31.07 (± 6.74)	t = −0.271	0.786
Bun, mg/dL, Mean (± SD)	26.98 (± 21.20)	24.29 (± 18.63)	40.94 (± 27.37)	t'= −14.779	< 0.001
Glucose, mg/dL, M (Q₁, Q₃)	134.69 (110–167)	135 (111–165)	134 (106.5–176.5)	W = 1,070,430	0.985
Bicarbonate, mEq/L, Mean (± SD)	23.34 (± 4.55)	23.56 (± 4.21)	22.22 (± 5.91)	t'= 5.497	< 0.001
Sodium, mEq/L, Mean (± SD)	137.70 (± 4.98)	137.60 (± 4.73)	138.24 (± 6.08)	t'= −2.510	0.012
Potassium, mEq/L, Mean (± SD)	4.40 (± 0.81)	4.41 (± 0.81)	4.36 (± 0.77)	t = 1.511	0.131
Chloride, mEq/L, M (Q₁, Q₃)	105 (101–108)	105 (102–109)	103 (98–108)	W = 1,246,849.5	< 0.001
PH, Mean (± SD)	7.37 (± 0.09)	7.38 (± 0.08)	7.34 (± 0.11)	t'= 8.174	< 0.001
PT, seconds, M (Q₁, Q₃)	15.48 (± 3.13)	15.22 (± 2.92)	16.80 (± 3.78)	t'= −10.015	< 0.001
PTT, seconds, Mean (± SD)	33.87 (± 8.44)	33.13 (± 8.03)	37.70 (± 9.42)	t'= −11.525	< 0.001
Urine output, mL, Mean (± SD)	1846.34 (± 1189.31)	1962.31 (± 1178.40)	1245.54 (± 1057.98)	t'= 15.431	< 0.001
Ventilation, n (%)				χ^2^ = 10.913	0.001
No	191 (4.81)	177 (5.31)	14 (2.18)		
Yes	3783 (95.19)	3154 (94.69)	629 (97.82)		
Vasopressor, n (%)				χ^2^ = 38.820	< 0.001
No	1668 (41.97)	1470 (44.13)	198 (30.79)		
Yes	2306 (58.03)	1861 (55.87)	445 (69.21)		
RRT, n (%)				χ^2^ = 131.583	< 0.001
No	3634 (91.44)	3121 (93.7)	513 (79.78)		
Yes	340 (8.56)	210 (6.3)	130 (20.22)		
Opiates drug, n (%)				χ^2^ = 4.258	0.039
No	1074 (27.03)	922 (27.68)	152 (23.64)		
Yes	2900 (72.97)	2409 (72.32)	491 (76.36)		
Propofol drug, n (%)				χ^2^ = 87.592	< 0.001
No	1497 (37.67)	1149 (34.49)	348 (54.12)		
Yes	2477 (62.33)	2182 (65.51)	295 (45.88)		
Midazolam drug, n (%)				χ^2^ = 113.078	< 0.001
No	3091 (77.78)	2694 (80.88)	397 (61.74)		
Yes	883 (22.22)	637 (19.12)	246 (38.26)		
Dexmedetomidine drug, n (%)				χ^2^ = 2.177	0.140
No	3337 (83.97)	2784 (83.58)	553 (86)		
Yes	637 (16.03)	547 (16.42)	90 (14)		
Cephalosporins, n (%)				χ^2^ = 11.370	0.001
No	1273 (32.03)	1030 (30.92)	243 (37.79)		
Yes	2701 (67.97)	2301 (69.08)	400 (62.21)		
Penicillins, n (%)				χ^2^ = 92.304	< 0.001
No	3041 (76.52)	2644 (79.38)	397 (61.74)		
Yes	933 (23.48)	687 (20.62)	246 (38.26)		
Carbapenems, n (%)				χ^2^ = 91.651	< 0.001
No	3653 (91.92)	3123 (93.76)	530 (82.43)		
Yes	321 (8.08)	208 (6.24)	113 (17.57)		
Quinolones, n (%)				χ^2^ = 27.565	< 0.001
No	3349 (84.27)	2852 (85.62)	497 (77.29)		
Yes	625 (15.73)	479 (14.38)	146 (22.71)		
Macrolides, n (%)				χ^2^ = 23.435	< 0.001
No	3785 (95.24)	3197 (95.98)	588 (91.45)		
Yes	189 (4.76)	134 (4.02)	55 (8.55)		
Metronidazole, n (%)				χ^2^ = 157.002	< 0.001
No	3312 (83.34)	2885 (86.61)	427 (66.41)		
Yes	662 (16.66)	446 (13.39)	216 (33.59)		
Aminoglycosides, n (%)				χ^2^ = 10.528	0.001
No	3928 (98.84)	3301 (99.1)	627 (97.51)		
Yes	46 (1.16)	30 (0.9)	16 (2.49)		
Polypeptides, n (%)				χ^2^ = 173.898	< 0.001
No	1666 (41.92)	1548 (46.47)	118 (18.35)		
Yes	2308 (58.08)	1783 (53.53)	525 (81.65)		
Tetracyclines, n (%)				-	0.368
No	3953 (99.47)	3315 (99.52)	638 (99.22)		
Yes	21 (0.53)	16 (0.48)	5 (0.78)		
Lincosamides, n (%)				χ^2^ = 0.533	0.466
No	3893 (97.96)	3266 (98.05)	627 (97.51)		
Yes	81 (2.04)	65 (1.95)	16 (2.49)		
Antifungal, n (%)				χ^2^ = 45.388	< 0.001
No	3745 (94.24)	3176 (95.35)	569 (88.49)		
Yes	229 (5.76)	155 (4.65)	74 (11.51)		
Follow-up time, days, Mean (± SD)	26.75 (± 7.95)	30.00 (± 0.00)	9.94 (± 7.34)	t'= 69.340	< 0.001
Number of encephalopathy related antibiotics use, n (%)				χ^2^ = 241.765	< 0.001
0	326 (8.2)	318 (9.55)	8 (1.24)		
1	2426 (61.05)	2147 (64.46)	279 (43.39)		
2	787 (19.8)	573 (17.2)	214 (33.28)		
≥ 3	435 (10.95)	293 (8.8)	142 (22.08)		

*SD* Standard Deviation, *M* Median, *Q₁* 1 st Quartile, *Q₃* 3 st Quartile, *t* Student's t test, *t'* Satterthwaite t test, *W* Wilcoxon rank sum test, *χ*^*2*^ Chi-square test,—Fisher's exact test, *ICU* intensive care unit, *AKI* acute kidney injury, *SOFA* Sequential Organ Failure Assessment, *GCS* Glasgow Coma Scale, *CCI* Charlson comorbidity index, *SBP* systolic blood pressure, *DBP* diastolic blood pressure, *RDW* red blood cell distribution width, *WBC* white blood cell count, *PT* prothrombin time, *PTT* partial thromboplastin time, *RRT* renal replacement therapy

### The relationship between the utilization of antibiotics either alone or in combination and the 30-day mortality risk in patients diagnosed with SAE

Table 2 shows the relationship between the utilization of antibiotics and the 30-day mortality risk in patients diagnosed with SAE. The use of penicillins (HR: 1.32, 95% CI: 1.11–1.58, *P* = 0.002), macrolides (HR: 1.50, 95% CI: 1.13–2.00, *P* = 0.005), and metronidazole (HR: 1.32, 95% CI: 1.11–1.57, *P* = 0.002) were associated with a higher risk of 30-day mortality in patients diagnosed with SAE. Conversely, the analysis did not reveal any statistical associations between the use of cephalosporins (HR: 0.97, 95% CI: 0.82–1.15, *P* = 0.740), carbapenems (HR: 1.13, 95% CI: 0.90–1.41, *P* = 0.284), quinolones (HR: 1.12, 95% CI: 0.93–1.36, *P* = 0.234) and the 30-day mortality risk in patients diagnosed with SAE.

**Table 2:** The relationship between the utilization of different antibiotics and the subsequent 30-day mortality risk in patients diagnosed with SAE

		Model 1		Model 2	
Variables	N (%)	HR (95% CI)	*P*	HR (95% CI)	*P*
Cephalosporins^a^					
No	1273 (32.03)	Ref		Ref	
Yes	2701 (67.97)	0.75 (0.64–0.88)	< 0.001	0.97 (0.82–1.15)	0.740
Penicillins^b^					
No	3041 (76.52)	Ref		Ref	
Yes	933 (23.48)	2.19 (1.86–2.56)	< 0.001	1.32 (1.11–1.58)	0.002
Carbapenems^c^					
No	3653 (91.92)	Ref		Ref	
Yes	321 (8.08)	2.69 (2.19–3.29)	< 0.001	1.13 (0.90–1.41)	0.284
Quinolones^d^					
No	3349 (84.27)	Ref		Ref	
Yes	625 (15.73)	1.63 (1.36–1.96)	< 0.001	1.12 (0.93–1.36)	0.234
Macrolides^e^					
No	3785 (95.24)	Ref		Ref	
Yes	189 (4.76)	2.02 (1.53–2.66)	< 0.001	1.50 (1.13–2.00)	0.005
Metronidazole^f^					
No	3312 (83.34)	Ref		Ref	
Yes	662 (16.66)	2.85 (2.42–3.35)	< 0.001	1.32 (1.11–1.57)	0.002

*HR* Hazard ratio, *CI* Confidence intervals, *Ref* reference, *SAE* sepsis associated encephalopathy

Model 1was unadjusted model

^a^Model 2 adjusted for age, race, AKI, SOFA, CCI, weight, heart rate, respiratory rate, SpO_2_, RDW, bun, sodium, chloride, PH, PTT, Urine output, ventilation, vasopressor, opiates drug, propofol drug, cephalosporin other drug

^b^Model 2 adjusted for age, race, AKI, SOFA, CCI, weight, heart rate, respiratory rate, SpO_2_, RDW, bun, sodium, chloride, PH, PTT, urine output, ventilation, vasopressor, opiates drug, propofol drug, penicillin other drug

^c^Model 2 adjusted for age, race, AKI, SOFA, CCI, weight, heart rate, respiratory rate, SpO_2_, RDW, Bun, sodium, chloride, PH, PTT, urine output, ventilation, vasopressor, opiates drug, propofol drug, carbapenem other drug

^d^Model 2 adjusted for age, race, AKI, SOFA, CCI, weight, heart rate, respiratory rate, SpO_2_, RDW, bun, sodium, chloride, PH, PTT, urine output, ventilation, vasopressor, opiates drug, propofol drug, quinolone other drug

^e^Model 2 adjusted for age, race, AKI, SOFA, CCI, weight, heart rate, respiratory rate, SpO_2_, RDW, bun, sodium, chloride, PH, PTT, urine output, ventilation, vasopressor, opiates drug, propofol drug, macrolide other drug

^f^Model 2 adjusted for age, race, AKI, SOFA, CCI, weight, heart rate, respiratory rate, SpO_2_, RDW, bun, sodium, chloride, PH, PTT, urine output, ventilation, vasopressor, opiates drug, propofol drug, metronidazole other drug

Regarding the association between a single antibiotic and the 30-day mortality risk in patients diagnosed with SAE (Supplementary Table 2), a lower 30-day mortality risk associated with cephalosporins use was observed (HR: 0.70, 95% CI: 0.55–0.87, *P* = 0.002). However, there was a higher mortality risk associated with penicillins use compared to non-use (HR: 1.33, 95% CI: 1.06–1.66, P = 0.013) in patients diagnosed with SAE. In addition, the results did not reach statistical significance between the association of the uses of carbapenems (HR: 1.41, 95% CI: 0.92–2.15, *P* = 0.111), quinolones (HR: 1.26, 95% CI: 0.77–2.07, *P* = 0.350), macrolides (*P* = 0.987), and metronidazole (HR: 1.87, 95% CI: 0.92–3.81, *P* = 0.082) with the 30-day mortality risk in patients diagnosed with SAE.

### The relationship between the number of six antibiotic classes used and the 30-day mortality risk in patients diagnosed with SAE

**Fig. 2:**
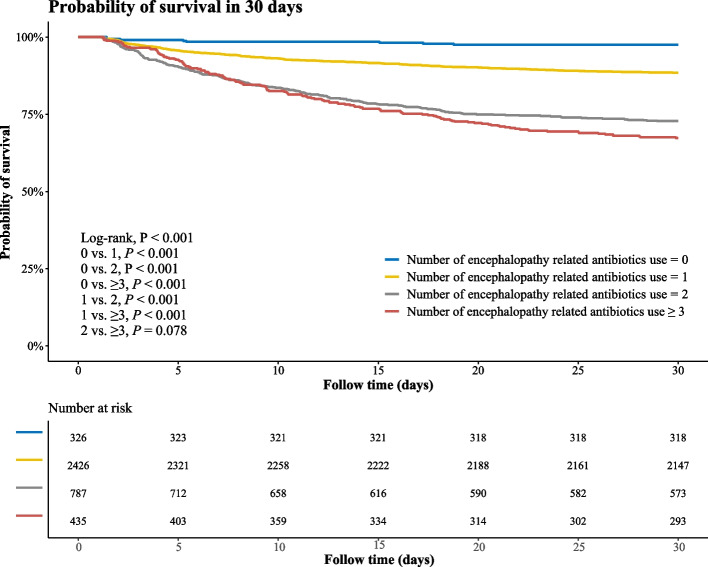
Kaplan–Meier curve of 30-day survival in patients who did and didn’t use the six classes of antibiotic

Compared to non-use of these six antibiotics, the utilization of one (HR: 3.36, 95% CI: 1.66–6.80,* P* = 0.001), two (HR: 3.98, 95% CI: 1.95–8.12, *P* < 0.001), or more than three antibiotic classes (HR: 4.34, 95% CI: 2.11–8.94,* P* < 0.001) from these six different classes were associated with an increased risk of 30-day mortality in patients with SAE. The relationship between the number of six antibiotic classes used and the subsequent 30-day mortality risk in patients diagnosed with SAE is presented in Table 3. The Kaplan–Meier curve (Fig. 2) shows that patients who did not use the six classes of antibiotic had the highest 30-day survival.

**Table 3:** The relationship between the number of six antibiotic species used and the subsequent 30-day mortality risk in patients diagnosed with SAE

		Model 1		Model 2	
Variables	N (%)	HR (95% CI)	*P*	HR (95% CI)	*P*
Number of encephalopathy related antibiotics use					
0	326 (8.20)	Ref		Ref	
1	2426 (61.05)	4.92 (2.44–9.93)	< 0.001	3.36 (1.66–6.80)	0.001
2	787 (19.80)	12.79 (6.31–25.91)	< 0.001	3.98 (1.95–8.12)	< 0.001
≥ 3	435 (10.95)	15.46 (7.59–31.52)	< 0.001	4.34 (2.11–8.94)	< 0.001

*HR* Hazard ratio, *CI* Confidence intervals, *Ref* reference, *SAE* sepsis associated encephalopathy

Model 1 was unadjusted model; Model 2 adjusted for age, race, AKI, SOFA, CCI, weight, heart rate, respiratory rate, SPO2, RDW, bun, sodium, chloride, PH, PTT, urine output, ventilation, vasopressor, opiates drug, propofol drug

### Subgroup analyses of the relationship between the number of six antibiotic classes used and the subsequent 30-day mortality risk in patients diagnosed with SAE

**Fig. 3:**
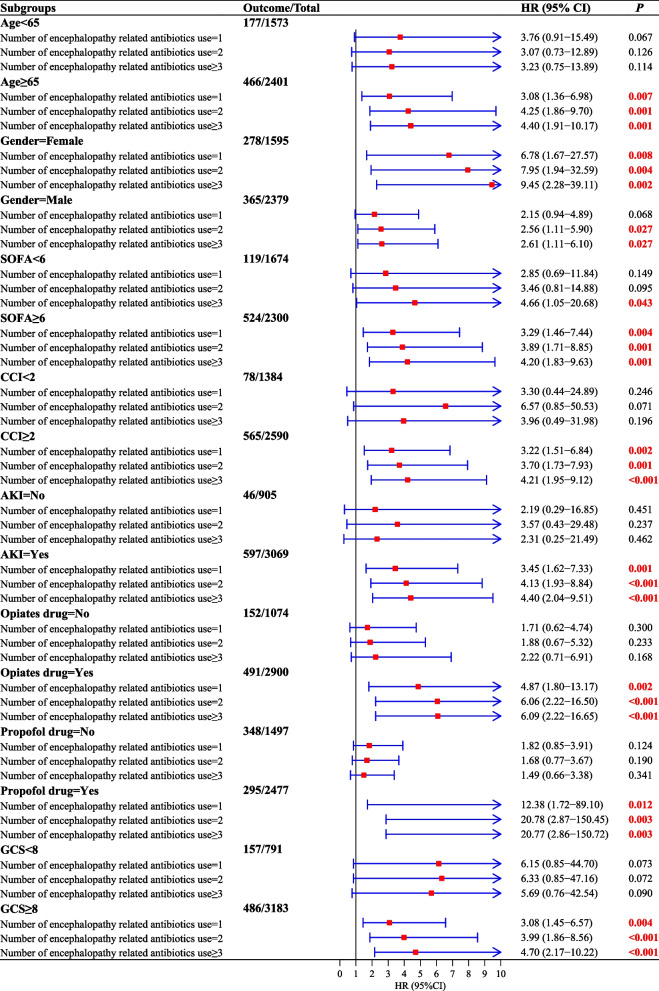
Subgroup analyses of the relationship between the number of six antibiotic classes used and the subsequent 30-day mortality risk in patients diagnosed with SAE

In the subset of SAE patients who were aged 65 or older, the use of one (HR: 3.08, 95% CI: 1.36–6.98, *P* = 0.007), two (HR: 4.25, 95% CI: 1.86–9.70, *P* = 0.001), and three or more (HR: 4.40, 95% CI: 1.91–10.17, *P* = 0.001) types of antibiotics from these six classes were related to an elevated risk of 30-day mortality. However, in the cohort of SAE patients below the age of 65, there was no statistically significant association between the use of one, two, and three or more types of antibiotics from these six classes and increased 30-day mortality risk. In the female population with SAE, the use of one (HR: 6.78, 95% CI: 1.67–27.57, *P* = 0.008), two (HR: 7.95, 95% CI: 1.94–32.59, *P* = 0.004), and three or more (HR: 9.45, 95% CI: 2.28–39.11, *P* = 0.002) types of antibiotic from these six classes was associated with an increased risk of 30-day mortality. Conversely, in the male SAE population, the use of two (HR: 2.56, 95% CI: 1.11–5.90, *P* = 0.027) and three or more (HR: 2.61, 95% CI: 1.11–6.10, *P* = 0.027) antibiotic classes from these six classes were linked to a higher 30-day mortality risk. The use of one, two, and three or more antibiotic classes was associated with an increased risk of 30-day mortality in SAE patients with SOFA > 6, CCI > 2, in those who had AKI, in those who used opiates, and propofol. For patients with GCS ≥ 8, there was a relationship where the use of an increasing number of antibiotics was significantly associated with a higher risk of mortality in the SAE population (Fig. 3).

## Discussion

The 30-day mortality rate of SAE patients observed in our study aligns with similar patient populations. For instance, a study utilizing the MIMIC-IV database reported a 30-day mortality rate of 17.62% among patients with SAE [27]. Another investigation found a mortality rate of 15.6% in patients with SAE after ICU admission in a comparable cohort [28]. The findings from our study indicate a significant increase in the 30-day mortality risk for patients with SAE who are treated with penicillins, macrolides, and metronidazole. In contrast, the use of cephalosporins, carbapenems, and quinolones did not demonstrate a statistically significant link to the 30-day mortality risk in SAE patients. This study further indicates that when considering the use of an antibiotic alone, cephalosporins are associated with a lower mortality risk, and penicillins with a higher risk, compared to no use. The associations with carbapenems, quinolones, macrolides, and metronidazole do not reach statistical significance when analyzing their impact alone. In addition, a greater number of antibiotic types used in the treatment of SAE patients was associated with an increased risk of 30-day mortality. In the subgroup of SAE patients who were 65 years of age or older, with a SOFA score greater than or equal to 6, a CCI score greater than or equal to 2, GCS ≥ 8, experiencing AKI, and those who were administered opiates or propofol, the number of the aforementioned six antibiotic classes was significantly associated with an increased 30-day mortality risk.

Beta-lactam antibiotics are first-line agents for the antimicrobial treatment of critically ill patients with sepsis or septic shock [29]. Despite being considered to have a safe therapeutic profile, there have been reports of neurotoxicity associated with beta-lactam antibiotics [30]. A study that enrolled 199 patients found an association between elevated beta-lactam concentrations and the deterioration of neurological function in ICU patients with sepsis [31]. In this study, the use of penicillins was found to be associated with increased 30-day mortality risk in patients with SAE. It may necessitate a re-evaluation of antibiotic stewardship programs to consider the potential association between specific antibiotics with patient outcomes, particularly in the context of SAE. Our findings also showed a significant increase in the 30-day mortality risk for patients with SAE who were treated with macrolides. A previous study concluded that rapamycin, a novel macrolide-class immunosuppressant, exerts complex effects on the brains of patients with Alzheimer's disease [32]. Carosi et al. found that in the later stages of Alzheimer's disease, when dementia becomes manifest, the lysosomal system in the brain is severely compromised, and treatment with rapamycin may exacerbate this damage [33]. Interestingly, in our study, when we analyzed the association between alone macrolides use and mortality in SAE patients did not reach statistical significance. This suggests that the observed associations may be influenced by confounding factors, such as underlying illness severity, rather than a direct causal effect of macrolides. Further research is warranted to elucidate the role of macrolides in SAE treatment, particularly in critically ill ICU patients. Previous research has found that metronidazole can cause adverse effects on the central nervous system, referred to as metronidazole-induced encephalopathy [14, 34]. A nested case–control study found that metronidazole is associated with an increased risk of adverse events affecting both peripheral and central nervous systems [35]. Our study is the first to identify an association between metronidazole use and the mortality of patients with SAE. However, this study demonstrates that when analyzing the association between the use of metronidazole alone and the mortality rates among patients with SAE, no significant association was observed. Our findings suggest that further research is warranted to elucidate the utilization of metronidazole in patients with SAE, particularly considering the diversity of antibiotic types and the individualized nature of clinical treatment.

Within our subgroup analysis, the use of the aforementioned six antibiotics classes in SAE patients aged over 65 years, with a SOFA score greater than or equal to 6, a CCI score greater than or equal to 2, suffering from AKI, or on opiates, and propofol, were related to an increased risk of 30-day mortality in SAE patients. These findings likely reflect the influence of underlying illness severity on mortality risk, as patients with these characteristics inherently face poorer prognoses independent of antibiotic exposure. For instance, older patients may be more vulnerable to SAE due to frailty [36]. Differences in pharmacokinetics and pharmacodynamics in older individuals compared to younger patients may impact the efficacy and safety of antibiotic therapy [37]. Similarly, higher SOFA and CCI scores, AKI, opioid use, and propofol use all indicate greater disease severity, which may contribute to suboptimal responses to treatment, thus elevating mortality risk [38]. Furthermore, in patients with GCS ≥ 8, an increasing number of encephalopathy-related antibiotics was significantly associated with higher mortality risk. The reason for this result may be that patients with GCS < 8 have more complex clinical presentations and higher disease severity, which often necessitates more aggressive interventions, including the use of a broader spectrum of antibiotics. This complexity and the intensity of treatment might obscure the relationship between antibiotic use and mortality risk in this subgroup.

The first strength of our study lies in its pioneering exploration of the association between various types of antibiotic use and the short-term mortality risk in patients with SAE. The large sample size of the study ensures robust and reliable results. Furthermore, the comprehensive multifactorial analysis, which incorporates a range of factors such as comorbidities, laboratory indicators, and treatment measures commonly found in the ICU clinical environment, allows for a more accurate assessment of the independent association between antibiotic use and mortality risk, controlling for potential confounders. Our study still has limitations. Firstly, as a retrospective cohort study, our research is inherently subject to the biases characteristic of this study type. The reliance on existing data and the inability to control for all potential confounders retrospectively may have introduced bias that could affect the interpretation of our findings. While we endeavored to adjust for known confounders, there may still be unmeasured variables that could influence the relationship between antibiotic use and short-term mortality risk in SAE patients. Secondly, the data used in our study were derived from a single medical center, which may limit the generalizability of our sample. The patient population, treatment protocols, and healthcare practices at our institution might not be representative of those in other centers. Therefore, our findings may not be directly applicable to different healthcare settings or diverse patient populations. To address this limitation, future research should involve large-scale, multicenter studies and randomized controlled trials to validate our results and to assess the external validity of our findings across different clinical settings and patient demographics. Thirdly, one of the primary limitations of our study is the lack of detailed information on minimum inhibitory concentration (MIC) levels within the MIMIC database. This absence precludes a precise analysis of the relationship between antibiotic dosing and therapeutic efficacy, which is particularly relevant for patients receiving loading doses to achieve the necessary MIC. Furthermore, the MIMIC database does not specifically distinguish between the administration of loading doses and standard dosing regimens. This limitation hinders our ability to accurately assess the association between loading doses with the patient outcomes, which could potentially influence the interpretation of our findings regarding the efficacy of different antibiotic classes. These limitations underscore the need for further research with more granular dosing data to better understand the role of loading doses in the treatment of SAE and to optimize antibiotic stewardship in intensive care settings. Fourthly, there are significant missing data regarding culture results in the database. This lack of information prevents us from accurately identifying whether patients had their medication classes adjusted based on culture results, which could influence the categorization of antibiotic exposure and the subsequent analysis of outcomes. This limitation underscores the need for future research to incorporate more comprehensive data on culture results to enhance the precision of antibiotic therapy analysis in critically ill patient.

## Conclusion

Our study suggests a potential link between the uses of penicillins, macrolides, and metronidazole and an increased risk of 30-day mortality in patients with SAE. However, it is important to note that further research is required to substantiate these findings and to fully understand the implications for antibiotic prescription practices in the context of SAE.

## Supplementary Information

Supplementary Material 1.

Supplementary Material 2.

## Data Availability

The datasets generated and/or analyzed during the current study are available in the MIMIC-IV database, https://mimic.mit.edu/docs/iv/.

## Abbreviations

SAE: Sepsis-associated encephalopathy
ICU: Intensive care unit
MIMIC: Medical Information Mart for Intensive Care
BIDMC: Beth Israel Deaconess Medical Center
AKI: Acute kidney injury
SBP: Systolic blood pressure
DBP: Diastolic blood pressure
SOFA: Sequential Organ Failure Assessment
GCS: Glasgow Coma Scale
CCI: Charlson comorbidity index
WBC: White blood cell count
BUN: Blood urea nitrogen
PT: Prothrombin time
PTT: Partial thromboplastin time
RRT: Renal replacement therapy
COPD: Chronic obstructive pulmonary disease
