# Supplementary material for: Relationship between antibiotic use and short-term risk of mortality in patients with sepsis-associated encephalopathy: a study based on the medical information mart for intensive care database
Source: BMC Infect Dis. 2025 Jul 1;25:858. doi: 10.1186/s12879-025-11139-3 (PMC12220782; doi:10.1186/s12879-025-11139-3)
Supplement: Supplementary file 1 — Supplementary Material 1. [file 12879_2025_11139_MOESM1_ESM.docx]

Supplementary Table 1 Identification of potential covariates

|  | Model 1 |  | Model 2 |  |
| --- | --- | --- | --- | --- |
| Variables | HR (95% CI) | *P* | HR (95% CI) | *P* |
| Age | 1.03 (1.02-1.03) | <0.001 | 1.02 (1.01-1.02) | <0.001 |
| Gender |  |  |  |  |
| Female | Ref |  |  |  |
| Male | 0.87 (0.74-1.01) | 0.069 |  |  |
| Race |  |  |  |  |
| Black | Ref |  | Ref |  |
| Others | 0.70 (0.50-0.99) | 0.044 | 0.80 (0.57-1.14) | 0.221 |
| Unknown | 1.27 (0.92-1.76) | 0.141 | 1.33 (0.96-1.84) | 0.089 |
| White | 0.67 (0.51-0.89) | 0.005 | 0.81 (0.61-1.07) | 0.138 |
| Chronic pulmonary disease |  |  |  |  |
| No | Ref |  |  |  |
| Yes | 1.51 (1.29-1.78) | <0.001 |  |  |
| AKI |  |  |  |  |
| No | Ref |  | Ref |  |
| Yes | 4.13 (3.06-5.58) | <0.001 | 1.81 (1.31-2.49) | <0.001 |
| Liver disease |  |  |  |  |
| No | Ref |  |  |  |
| Yes | 2.50 (2.05-3.04) | <0.001 |  |  |
| Anemia |  |  |  |  |
| No | Ref |  |  |  |
| Yes | 1.20 (0.96-1.51) | 0.104 |  |  |
| SOFA | 1.21 (1.19-1.23) | <0.001 | 1.09 (1.07-1.12) | <0.001 |
| CCI | 1.28 (1.25-1.32) | <0.001 | 1.13 (1.09-1.16) | <0.001 |
| Weight | 0.99 (0.98-0.99) | <0.001 | 0.99 (0.98-0.99) | <0.001 |
| Heart rate | 1.02 (1.01-1.02) | <0.001 | 1.01 (1.00-1.01) | <0.001 |
| SBP | 0.99 (0.99-1.00) | <0.001 |  |  |
| DBP | 1.00 (0.99-1.00) | 0.200 |  |  |
| Respiratory rate | 1.08 (1.07-1.10) | <0.001 | 1.04 (1.02-1.05) | <0.001 |
| SpO_2_ | 0.87 (0.86-0.89) | <0.001 | 0.97 (0.95-0.99) | 0.008 |
| Temperature | 1.06 (0.95-1.19) | 0.264 |  |  |
| RDW | 1.23 (1.21-1.26) | <0.001 | 1.12 (1.08-1.15) | <0.001 |
| Platelet | 1.00 (1.00-1.00) | 0.100 |  |  |
| WBC | 1.01 (1.00-1.01) | 0.008 |  |  |
| Hemoglobin | 0.96 (0.93-1.00) | 0.031 |  |  |
| Hematocrit | 1.00 (0.99-1.01) | 0.787 |  |  |
| Bun | 1.02 (1.02-1.02) | <0.001 | 1.00 (1.00-1.01) | 0.010 |
| Glucose | 1.00 (1.00-1.00) | 0.111 |  |  |
| Bicarbonate | 0.93 (0.92-0.95) | <0.001 |  |  |
| Sodium | 1.02 (1.01-1.04) | 0.003 | 1.03 (1.01-1.05) | 0.009 |
| Potassium | 0.93 (0.85-1.03) | 0.168 |  |  |
| Chloride | 0.96 (0.95-0.97) | <0.001 | 0.98 (0.96-0.99) | 0.003 |
| PH | 0.02 (0.01-0.03) | <0.001 | 0.41 (0.19-0.92) | 0.030 |
| PT | 1.14 (1.12-1.17) | <0.001 |  |  |
| PTT | 1.05 (1.04-1.06) | <0.001 | 1.02 (1.01-1.03) | <0.001 |
| Urineoutput | 1.00 (1.00-1.00) | <0.001 | 1.00 (1.00-1.00) | <0.001 |
| Ventilation |  |  |  |  |
| No | Ref |  | Ref |  |
| Yes | 2.40 (1.41-4.07) | 0.001 | 1.54 (0.89-2.65) | 0.122 |
| Vasopressor |  |  |  |  |
| No | Ref |  | Ref |  |
| Yes | 1.72 (1.46-2.04) | <0.001 | 1.23 (0.99-1.52) | 0.059 |
| RRT |  |  |  |  |
| No | Ref |  |  |  |
| Yes | 3.16 (2.61-3.83) | <0.001 |  |  |
| Opiates drug |  |  |  |  |
| No | Ref |  | Ref |  |
| Yes | 1.24 (1.03-1.49) | 0.020 | 1.65 (1.33-2.05) | <0.001 |
| Propofol drug |  |  |  |  |
| No | Ref |  | Ref |  |
| Yes | 0.47 (0.41-0.55) | <0.001 | 0.51 (0.42-0.61) | <0.001 |
| Midazolam drug |  |  |  |  |
| No | Ref |  |  |  |
| Yes | 2.37 (2.03-2.78) | <0.001 |  |  |
| Dexmedetomidine drug |  |  |  |  |
| No | Ref |  |  |  |
| Yes | 0.82 (0.66-1.03) | 0.085 |  |  |

Notes: AKI, acute kidney injury; SOFA, Sequential Organ Failure Assessment; CCI, Charlson comorbidity index; SBP, systolic blood pressure; DBP, diastolic blood pressure; RDW, red blood cell distribution width; WBC, white blood cell count; PT, prothrombin time; PTT, partial thromboplastin time; RRT, renal replacement therapy; HR: hazard ratio; CI: confidence intervals; Ref: reference;

Model 1was unadjusted model;

Model 2 was a multivariable model with covariates selected after bidirectional stepwise regression.
