# Supplementary material for: Relationship between antibiotic use and short-term risk of mortality in patients with sepsis-associated encephalopathy: a study based on the medical information mart for intensive care database
Source: BMC Infect Dis. 2025 Jul 1;25:858. doi: 10.1186/s12879-025-11139-3 (PMC12220782; doi:10.1186/s12879-025-11139-3)
Supplement: Supplementary file 2 — Supplementary Material 2. [file 12879_2025_11139_MOESM2_ESM.docx]

**Supplementary Table 2 Impact of only one group of antibiotic alone on the 30-day mortality risk in patients diagnosed with SAE**

| Variables | N (%) | Model 1 | | Model 2 | |
| --- | --- | --- | --- | --- | --- |
|  |  | HR (95% CI) | *P* | HR (95% CI) | *P* |
| Cephalosporins^a^ |  |  |  |  |  |
| No | 1273(41.72) | Ref |  | Ref |  |
| Yes | 1778(58.28) | 0.35 (0.28-0.44) | <0.001 | 0.70 (0.55-0.87) | 0.002 |
| Penicillins^b^ |  |  |  |  |  |
| No | 3041(88.04) | Ref |  | Ref |  |
| Yes | 413(11.96) | 2.04 (1.64-2.53) | <0.001 | 1.33 (1.06-1.66) | 0.013 |
| Carbapenems^c^ |  |  |  |  |  |
| No | 3653(98.09) | Ref |  | Ref |  |
| Yes | 71(1.91) | 2.61 (1.74-3.93) | <0.001 | 1.41 (0.92-2.15) | 0.111 |
| Quinolones^d^ |  |  |  |  |  |
| No | 3349(96.74) | Ref |  | Ref |  |
| Yes | 113(3.26) | 0.99 (0.61-1.61) | 0.981 | 1.26 (0.77-2.07) | 0.350 |
| Macrolides^e^ |  |  |  |  |  |
| No | 3785(99.45) | Ref |  | Ref |  |
| Yes | 21(0.55) | 0.00 (0.00-Inf) | 0.988 | 0.00 (0.00-Inf) | 0.987 |
| Metronidazole^f^ |  |  |  |  |  |
| No | 3312(99.10) | Ref |  | Ref |  |
| Yes | 30(0.90) | 2.26 (1.13-4.56) | 0.022 | 1.87 (0.92-3.81) | 0.082 |

Notes: HR: Hazard ratio; CI: Confidence intervals; Ref: reference; SAE, sepsis associated encephalopathy.
Model 1was unadjusted model;

a: Model 2 adjusted for age, race, AKI, SOFA, CCI, weight, heart rate, systolic, respiratory rate, SpO2, RDW, hemoglobin, sodium, chloride, PH, PTT, urine output, ventilation, RRT, propofol drug, midazolam drug, and dexmedetomidine drug.

b: Model 2 adjusted for age, race, AKI, SOFA, CCI, weight, heart rate, respiratory rate, SpO_2_, RDW, BUN, chloride, PH, PTT, urine output, ventilation, vasopressor, RRT, propofol drug, and midazolam drug.

c: Model 2 adjusted for age, gender, race, AKI, SOFA, CCI, weight, heart rate, respiratory rate, SpO_2_, RDW, BUN, sodium, chloride, PH, PTT, urine output, ventilation, vasopressor, RRT, propofol drug, midazolam drug, and dexmedetomidine drug.

d: Model 2 adjusted for age, gender, race, AKI, SOFA, CCI, weight, heart rate, systolic, respiratory rate, SpO_2_, RDW, hemoglobin, BUN, bicarbonate, sodium, chloride, PTT, urine output, ventilation, RRT, propofol drug, and midazolam drug.

e: Model 2 adjusted for age, race, AKI, SOFA, CCI, weight, heart rate, respiratory rate, SpO_2_, RDW, Hemoglobin, BUN, sodium, chloride, PH, PTT, urine output, ventilation, vasopressor, RRT, propofol drug, midazolam drug, and dexmedetomidine drug.

f: Model 2 adjusted for age, race, AKI, SOFA, CCI, weight, heart rate, respiratory rate, RDW, hemoglobin, BUN, sodium, chloride, PTT, urine output, ventilation, RRT, propofol drug, and midazolam drug.
